# Head-to-head comparison of relative cerebral blood flow derived from dynamic [^18^F]florbetapir and [^18^F]flortaucipir PET in subjects with subjective cognitive decline

**DOI:** 10.1186/s13550-023-01041-x

**Published:** 2023-10-27

**Authors:** Hayel Tuncel, Denise Visser, Tessa Timmers, Emma E. Wolters, Rik Ossenkoppele, Wiesje M. van der Flier, Bart N. M. van Berckel, Ronald Boellaard, Sandeep S. V. Golla

**Affiliations:** 1 Department of Radiology and Nuclear Medicine, Amsterdam Neuroscience, Vrije Universiteit Amsterdam, Amsterdam UMC, De Boelelaan 1117, 1081 HV Amsterdam, The Netherlands; 2 Department of Neurology, Alzheimer Center Amsterdam, Vrije Universiteit Amsterdam, Amsterdam UMC, Amsterdam, The Netherlands; 3 Clinical Memory Research Unit, Lund University, Lund, Sweden; 4 Department of Epidemiology and Biostatistics, Vrije Universiteit Amsterdam, Amsterdam UMC, Amsterdam, The Netherlands

**Keywords:** PET, Relative cerebral blood flow, [^18^F]florbetapir, [^18^F]flortaucipir, SCD

## Abstract

**Background:**

Dynamic PET imaging studies provide accurate estimates of specific binding, but also measure the relative tracer delivery (R_1_), which is a proxy for relative cerebral blood flow (rCBF). Recently, studies suggested that R_1_ obtained from different tracers could be used interchangeably and is irrespective of target tissue. However, the similarities or differences of R_1_ obtained from different PET tracers still require validation. Therefore, the goal of the current study was to compare R_1_ estimates, derived from dynamic [^18^F]florbetapir (amyloid) and [^18^F]flortaucipir (tau) PET, in the same subjects with subjective cognitive decline (SCD).

**Results:**

Voxel-wise analysis presented a small cluster (1.6% of the whole brain) with higher R_1_ values for [^18^F]flortaucipir compared to [^18^F]florbetapir in the Aβ-negative group. These voxels were part of the hippocampus and the left middle occipital gyrus. In part of the thalamus, midbrain and cerebellum, voxels (2.5% of the whole brain) with higher R_1_ values for [^18^F]florbetapir were observed. In the Aβ-positive group, a cluster (0.2% of the whole brain) of higher R_1_ values was observed in part of the hippocampus, right parahippocampal gyrus and in the left sagittal stratum for [^18^F]flortaucipir compared to [^18^F]florbetapir. Furthermore, in part of the thalamus, left amygdala, midbrain and right parahippocampal gyrus voxels (0.4% of the whole brain) with higher R_1_ values for [^18^F]florbetapir were observed. Despite these differences, [^18^F]florbetapir R_1_ had high correspondence with [^18^F]flortaucipir R_1_ across all regions of interest (ROIs) and subjects (Aβ−:*r*^2^ = 0.79, slope = 0.85, ICC = 0.76; Aβ+:* r*^2^ = 0.87, slope = 0.93, ICC = 0.77).

**Conclusion:**

[^18^F]flortaucipir and [^18^F]florbetapir showed similar R_1_ estimates in cortical regions. This finding, put together with previous studies, indicates that R_1_ could be considered a surrogate for relative cerebral blood flow (rCBF) in the cortex and may be used interchangeably, but with caution, regardless of the choice of these two tracers.

## Introduction

Alzheimer’s disease (AD) is a progressive neurodegenerative disorder characterized by accumulation of intracellular neurofibrillary tangles (NFTs) and extracellular amyloid-β (Aβ) plaques in the brain [1]. The accumulation causes neuropathological changes, such as synaptic and neuronal cell death and decreased cognitive function. Aβ plaques are believed to be one of the hallmarks driving AD pathogenesis and are used for diagnosis; however, there is evidence that vascular and metabolic factors are involved in the development and progression of AD as well [2]. Previous imaging studies have shown that reduced cerebral blood (CBF) and cerebral metabolic rates of glucose in the brain could serve as possible biomarkers of AD [3, 4]. More importantly, these biomarkers are associated with cognitive decline and conversion to AD; therefore, they can be used to identify clinical drug trial participants who are in a milder and earlier phase of the disease [5, 6].

Radiolabelled water, also called [^15^O]H_2_O, has a linear relationship with CBF [7]. Hence, the gold standard for measurement of CBF is [^15^O]H_2_O positron emission tomography (PET). However, the short half-life of ^15^O makes it difficult to use it in clinical practice. Moreover, the test–retest repeatability (TRT) of K_1_ obtained for [^15^O]H_2_O is quite low (9.7 ± 10.4%) [8], which is inconvenient for longitudinal or drug intervention studies. A way to measure CBF is arterial spin labelling of magnetic resonance imaging (MRI) [9] which has a good correspondence with [^15^O]H_2_O PET, but also moderate reliability over time (ICC = 0.63–0.74) [10, 11]. However, previous studies have presented R_1_ obtained from a dynamic PET scan as a surrogate of relative cerebral blood flow (rCBF) [12–17]. R_1_ represents the ratio of tracer influx in target regions relative to the reference region and can be obtained from the same PET (dynamic) scan without requirement of any additional scans. Several studies have shown that R_1_ estimates obtained with some amyloid and tau tracers such as [^11^C]PIB, [^18^F]THK5317 and [^18^F]AV45 are strongly correlated with CBF obtained from [^15^O]H_2_O PET and metabolic activity derived from [^18^F]FDG PET [12, 13, 16, 17]. Furthermore, the TRT of [^18^F]florbetapir and [^18^F]flortaucipir R_1_ is higher when compared to the TRT of [^15^O]H_2_O, 2.1 ± 1.1% and 1.8 ± 1.3%, respectively [8, 18]. This suggests that dynamic [^18^F]florbetapir and [^18^F]flortaucipir PET scans do not only provide quantitative information of amyloid-β and tau pathology but also yield estimates on rCBF and ultimately circumvent the need for an additional [^15^O]H_2_O, [^18^F]FDG scan or MRI for AD patients. This approach would be highly useful in the clinic, since it results in decreased costs, lower radiation dose and increased patient comfort.

A study by Rodriguez-Vieitez [13] investigated the comparability of [^18^F]THK5317- and [^11^C]PIB-derived rCBF in 11 mild cognitive impairment subjects and 8 AD patients. The researchers showed a high correlation (*r* = 0.90) in R_1_ between the two tracers. Moreover, another recent study found [19] a strong correlation between [^18^F]MK6240 and [^11^C]PiB R_1_ (*r* = 0.93). These findings suggest that R_1_ obtained from different tracers could be used interchangeably and is irrespective of target tissue. Although a few studies have indicated that R_1_ can be used as a surrogate of rCBF, the similarities or differences of R_1_ estimates obtained from different PET tracers still require validation. It is of importance to determine whether the outcome of R_1_ is similar between different tracers, since rCBF should not depend on the choice of the tracer. Therefore, the aim of the current study was to compare the R_1_ estimates derived from dynamic [^18^F]florbetapir and [^18^F]flortaucipir PET scans and assess whether R_1_ can serve as a tracer-independent surrogate of rCBF, in case of these specific tracers. To this end, a head-to-head comparison was made using [^18^F]florbetapir and [^18^F]flortaucipir PET scans obtained from the same subjects with subjective cognitive decline (SCD).

## Methods

### Participants

Fifty SCD subjects from the Amsterdam Dementia Cohort [20, 21] and Subjective Cognitive ImpairmENt Cohort (SCIENCe) study [22] were included. A standardized dementia screening was performed for all subjects, including medical history, extensive neuropsychological assessment, physical and neurological examination, lumbar puncture, blood tests, electroencephalography and brain MRI. The subjects were labelled as SCD, based on self-reported cognitive complaints, without objective impairment on neuropsychological or neurological tasks or brain damage as visualized by MRI [23]. Nineteen out of 50 SCD subjects were classified as amyloid positive as evidenced by substantial Aß pathology after visual assessment of [^18^F]florbetapir Aß-PET (SUVr_50–70 min_) scans (with grey matter (GM) cerebellum as the reference region) by an experienced nuclear medicine physician. Exclusion criteria were: significant cerebrovascular disease as assessed by MRI, major traumatic brain injury, major psychiatric or neurological disorders (other than AD) and recent substance abuse. The study was approved by the Medical Ethics Review Committee of Amsterdam UMC. All subjects signed written informed consent prior to study participation. All procedures performed were in accordance with the ethical standards of the institutional research committee and with the 1975 Helsinki Declaration and its later amendments.

### Data acquisition and processing

All subjects underwent a dynamic [^18^F]florbetapir and [^18^F]flortaucipir PET scan, both acquired on a Philips Ingenuity TF-64 PET/CT scanner, with a time period of 50.6 ± 34.7 days between both PET scans. The scan procedure for the [^18^F]florbetapir PET scans was as followed: first, following a low-dose computed tomography (CT) scan for attenuation corrections, a 90-min dynamic PET scan was obtained with 319 ± 25 MBq [^18^F]florbetapir injected activity. [^18^F]florbetapir PET scans were reconstructed using ordered subsets time of flight (BLOB-OS-TF) into a total of 22 frames (1 × 15, 3 × 5, 3 × 10, 4 × 60, 2 × 150, 2 × 300 and 7 × 600 s). The scan procedure for the [^18^F]flortaucipir PET scans consisted of two time windows of 60 and 50 min, respectively, with a 20-min break in between. Each time window was preceded by a low-dose CT for attenuation correction. The first time window of the PET scan was acquired simultaneously with a bolus injection 241 ± 11 MBq [^18^F]flortaucipir. Using VINCI software (Max Plank Institute, Cologne, Germany), the second time window of the PET scan was co-registered to the first time window. The PET list mode data were rebinned into a total of 29 frames (1 × 15, 3 × 5, 3 × 10, 4 × 60, 2 × 150, 2 × 300, 4 × 600 and 10 × 300 s), and raw data were reconstructed using 3D RAMLA. During the reconstruction of all PET scans, corrections for decay, dead time, normalization, attenuation, random coincidences and scatter were applied. All reconstructed PET images, for both tracers, had a matrix size of 128 × 128 × 90 and a voxel size of 2 × 2 × 2 mm^3^.

Furthermore, T1-weighted MRI scans were acquired for all subjects using a 3.0 T Philips Ingenuity Time-of-Flight PET/MR scanner (Philips medical systems, Best, the Netherlands) for structural information and brain tissue segmentation.

### Data analysis

The T1-weighted MRI scans were co-registered onto the corresponding PET images in Vinci software. Using PVElab [24] and Hammers template [25], volumes of interest were delineated on the co-registered MR images and superimposed on the PET scan to obtain regional time activity curves (TACs). PVElab utilizes a region of interest probability map created on the basis of a database of several subjects' T1-weighted MR images, where regions of interest (ROIs) have been pre-defined manually. The PET scan and the T1-weighted MRI scan (co-registered to PET) were used as input in PVElab for each subject separately. Within PVElab, these different T1-weighted MRI scans and associated pre-segmented templates are co-registered onto the MRI scan of interest. By this, a probability map of ROIs for the MRI scan of interest is obtained. Voxel-wise parametric images of R_1_ were generated using receptor parametric mapping (RPM, a basis function approach of simplified reference tissue model) with cerebellar GM as the reference region [26–28]. For this purpose, the entire duration of the PET scan was used. For regional analysis (in subject space), the following GM bilateral ROIs were produced a priori combining brain regions from the Hammers template [25]: frontal cortex, parietal cortex, temporal cortex, occipital cortex, thalamus, putamen, hippocampus, insula, brainstem and whole brain.

Voxel-wise analyses were performed to create average images of the subject groups with different amyloid status (Aβ negative and Aβ positive) and to explore differences in [^18^F]florbetapir and [^18^F]flortaucipir R_1_. For this purpose, Statistical Parametric Mapping (SPM) version 12 software (Welcome Trust Center for Neuroimaging, University College London, UK) was used. First, all native space parametric R_1_ images were warped to Montreal Neurological Institute (MNI152) space using the transformation matrixes derived from warping the co-registered MRI scans to MNI space. Warped images underwent quality control in order to avoid transformation errors. After warping, PET images were smoothed by a Gaussian filter of 8 mm FWHM over a 3D space to increase signal-to-noise ratio for statistical analysis.

### Statistical analyses

Paired samples t-tests were used for all the analyses (regional and voxel-wise) to compare R_1_ values from [^18^F]florbetapir and [^18^F]flortaucipir scans. A *p*-value below 0.001 (uncorrected for family-wise error (FWE), with cluster size > 25 voxels) was considered significant for voxel-wise analyses. Furthermore, a more conservative FWE rate Bonferroni correction (*p* < 0.05) was assessed. In addition to the voxel-wise analysis in SPM, an additional regional analysis was performed. For that, the output of the voxel-wise SPM analysis was used to obtain a mask. In this mask, all the significantly different voxels between the two tracers from the SPM analysis had a value of one and the rest of the voxels had a value of zero, so a binary mask was obtained. This mask was used to obtain the regional R_1_ values (i.e. a region for “significant voxels” and another for the “non-significant voxels”) for each subject and tracer. This entire procedure was performed in MNI space. Lastly, correlations coefficients (*r*^2^) were determined and an intraclass correlation coefficient (ICC) was calculated using an absolute agreement, two-way mixed-effects model for R_1_.

## Results

The current data did now show significant differences in age (*p* = 0.053) or MMSE (*p* = 0.40) between the Aβ-positive and Aβ-negative subject groups. Furthermore, no significant differences were observed in R_1_ between the Aβ-positive and Aβ-negative subjects in whole brain for [^18^F]flortaucipir (*p* = 0.15) and [^18^F]florbetapir (*p* = 0.51).

### *Comparison of *average *R*_*1*_*maps per tracer and amyloid status*

**Fig. 1:**
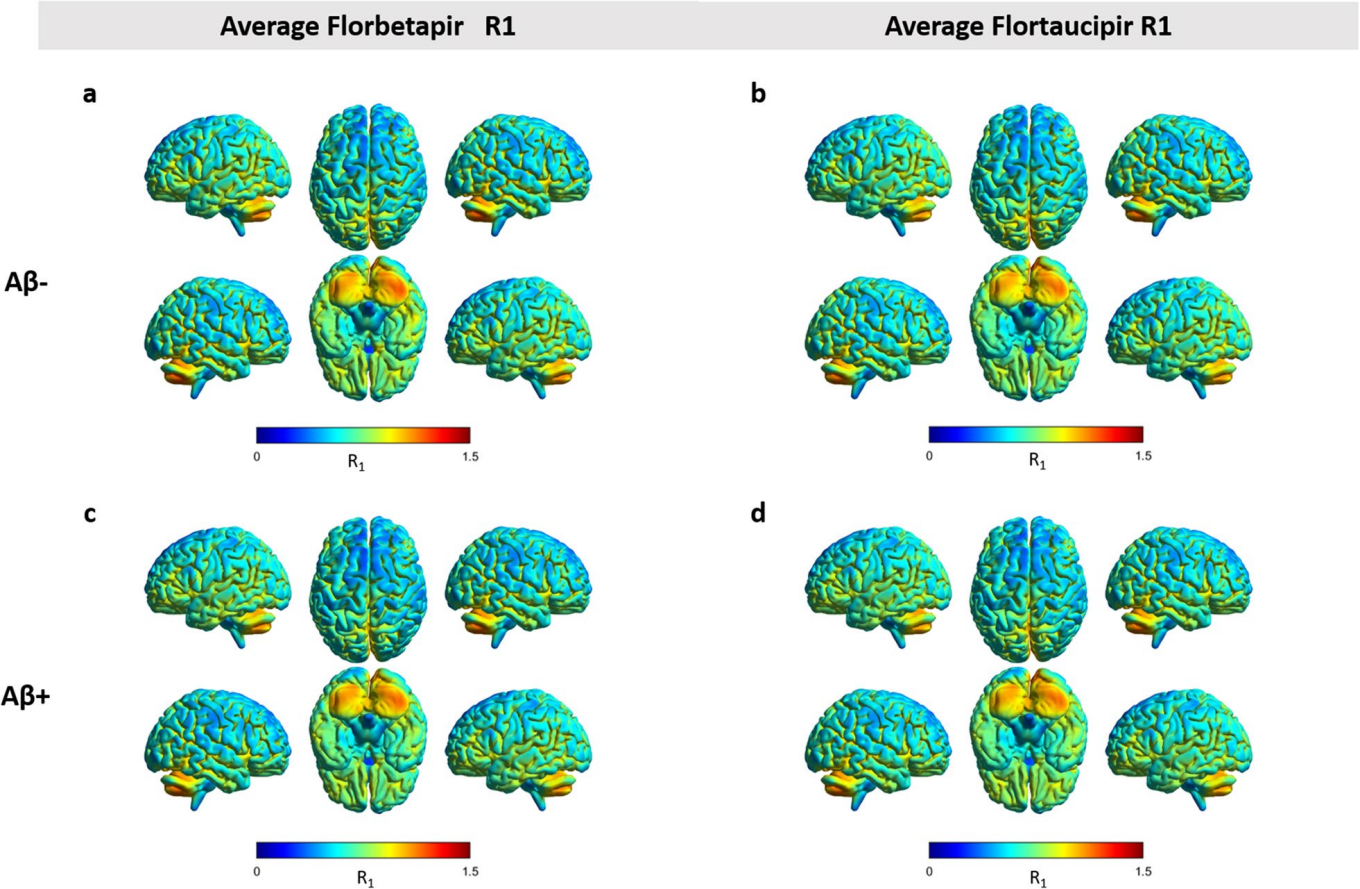
Average [^18^F]florbetapir and [^18^F]flortaucipir R_1_ images for all Aβ-negative (**a, b**) and Aβ-positive subjects (**c, d**)

No visual differences in the cortical regions between the average R_1_ images of [^18^F]florbetapir and [^18^F]flortaucipir were observed, irrespective of amyloid status (Fig. 1).

### ***Voxel-wise comparison of [***^***18***^***F]florbetapir and [***^***18***^***F]flortaucipir R***_***1***_*** images***

#### Aβ-negative group

**Fig. 2:**
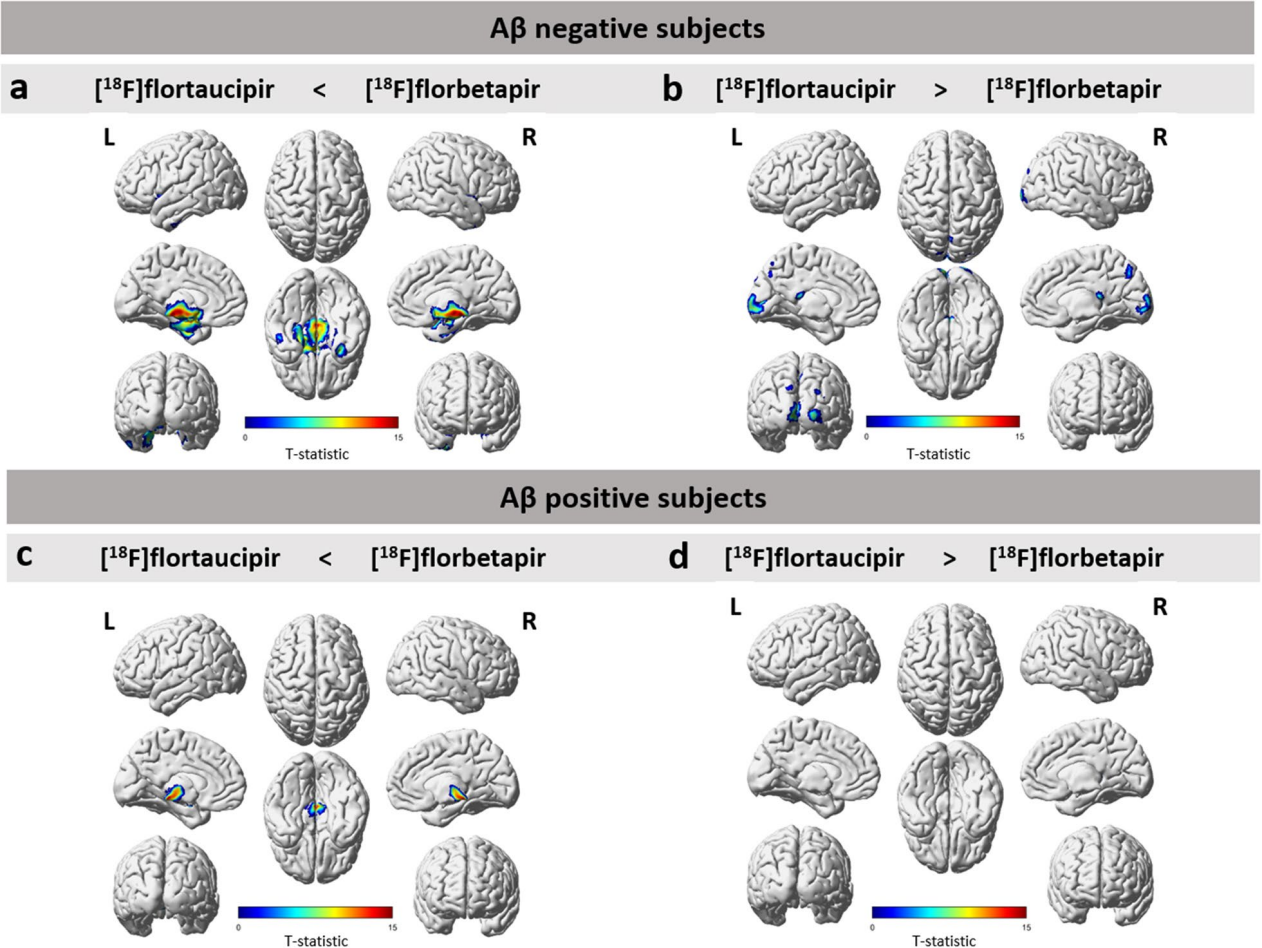
Results from voxel-wise analysis using SPM12 between R_1_ images of [^18^F]flortaucipir and [^18^F]florbetapir, separately for the Aβ-negative (**a, b**) and Aβ-positive group (**c, d**)

Voxel-wise analysis showed higher R_1_ values for [^18^F]florbetapir when compared to [^18^F]flortaucipir in a few clusters (2.5% of the whole brain) which were located in part of the thalamus (bilateral), midbrain (bilateral), left red nucleus, left entorhinal cortex, right hippocampus, left posterior insula, left hypothalamus, right fusiform gyrus, right nucleus accumbens, right medial temporal gyrus and small part of the cerebellum (bilateral) (Fig. 2a). Differences in these regions survived FWE correction. Furthermore, a few clusters (1.6% of the whole brain) showed higher R_1_ values in the hippocampus (bilateral) and the left middle occipital gyrus for [^18^F]flortaucipir compared to [^18^F]florbetapir (Fig. 2b). Differences in these areas too survived FWE correction.

#### Aβ-positive group

Voxel-wise analysis showed small significant clusters (0.4% of the whole brain), indicating higher R_1_ values for [^18^F]florbetapir when compared to [^18^F]flortaucipir. These clusters were mainly located in part of the thalamus (bilateral), left amygdala, midbrain (bilateral) and right parahippocampal gyrus (Fig. 2c). Even with FWE correction, these regions retained the significant differences. Furthermore, a very small cluster of significant voxels (0.2% of the whole brain) was observed for [^18^F]flortaucipir in the hippocampus (bilateral), right parahippocampal gyrus and in the left sagittal stratum (Fig. 2d). Differences in the hippocampus and stratum survived FWE correction.

#### Difference images

**Fig. 3:**
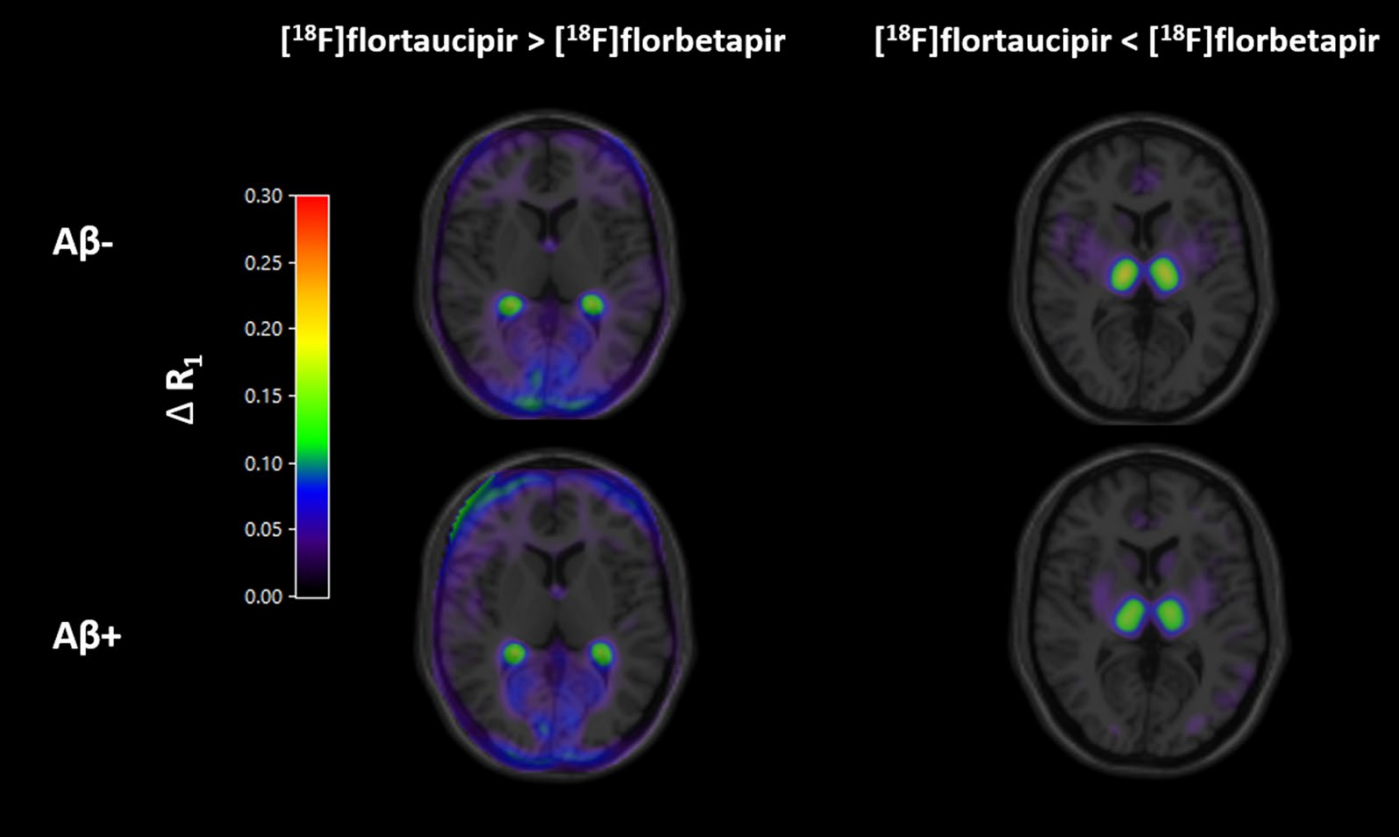
Average ΔR_1_ images superimposed with T1-weighted MRI scan in MNI space: average of the ΔR_1_ images in MNI space for different conditions ([^18^F]flortaucipir > [^18^F]florbetapir and, [^18^F]flortaucipir < [^18^F]florbetapir) separately for Aβ-negative and Aβ-positive subjects are illustrated

Average parametric RPM R_1_ difference images between [^18^F]flortaucipir and [^18^F]florbetapir are presented in Fig. 3, separately for Aβ-negative and Aβ-positive subjects.

### ***Regional comparisons between [***^***18***^***F]florbetapir and [***^***18***^***F]flortaucipir R***_1_*** images in MNI space***

**Fig. 4:**
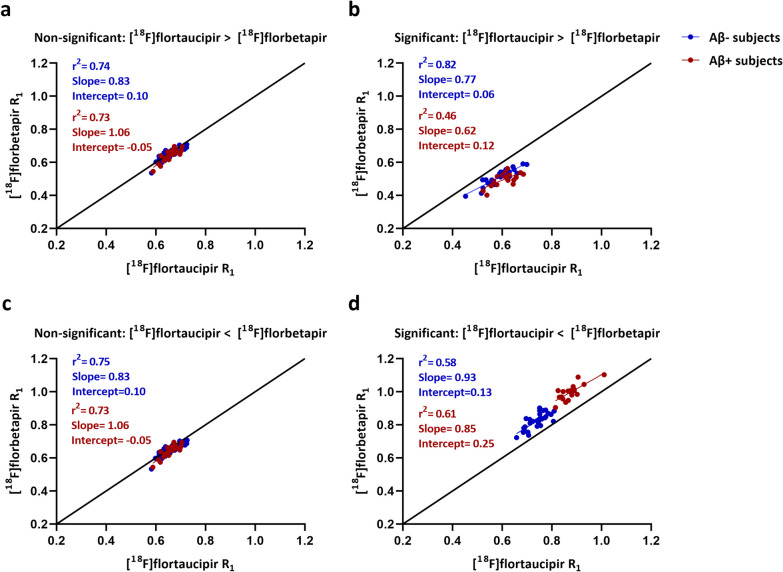
Correlations and slopes between [^18^F]flortaucipir R_1_ and [^18^F]florbetapir R_1_ for significant (**b, d**) and non-significant voxels (**a, c**). Two conditions are also illustrated in the figure: [^18^F]flortaucipir > [^18^F]florbetapir (**a, b**) and [^18^F]flortaucipir < [^18^F]florbetapir (**c, d**). Each dot in the figure represents a subject (blue dots represent amyloid-negative subjects and red dots represent amyloid-positive subjects). Significant: Voxels with significant differences from the voxel-wise analysis. Non-significant: Voxels with no significant differences from the voxel-wise analysis

For the voxels with no significant differences in R_1_ between the [^18^F]florbetapir and [^18^F]flortaucipir tracers, a good correspondence was observed irrespective of the amyloid status as illustrated in Fig. 4 (range *r*^2^: 0.73–0.75, slope: 0.83–1.06). Interestingly, even for the voxels with significant differences, a good correspondence (range *r*^2^: 0.46–0.82, slope: 0.62–0.93) was observed (Fig. 4). However, in this scenario, amyloid status seems to have a negative impact on the correspondence.

### *Regional [*^***18***^*F]florbetapir and [*^*18*^*F]flortaucipir R1 comparisons in native space*

**Fig. 5:**
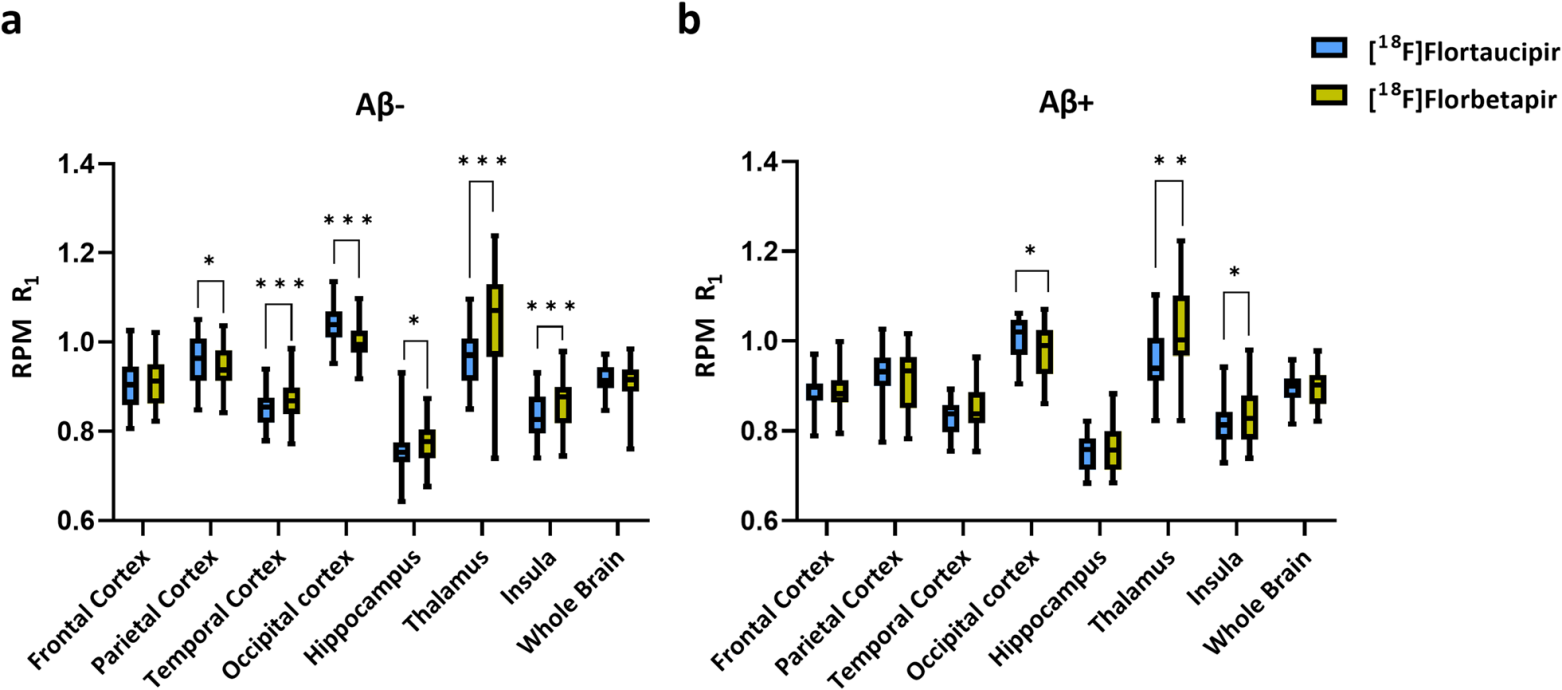
Bar plots illustrating the regional comparisons of *R*_1_ values (in native space) of [^18^F]flortaucipir and [^18^F]florbetapir, separately for Aβ-negative (**a**) and Aβ-positive (**b**) subjects

**Fig. 6:**
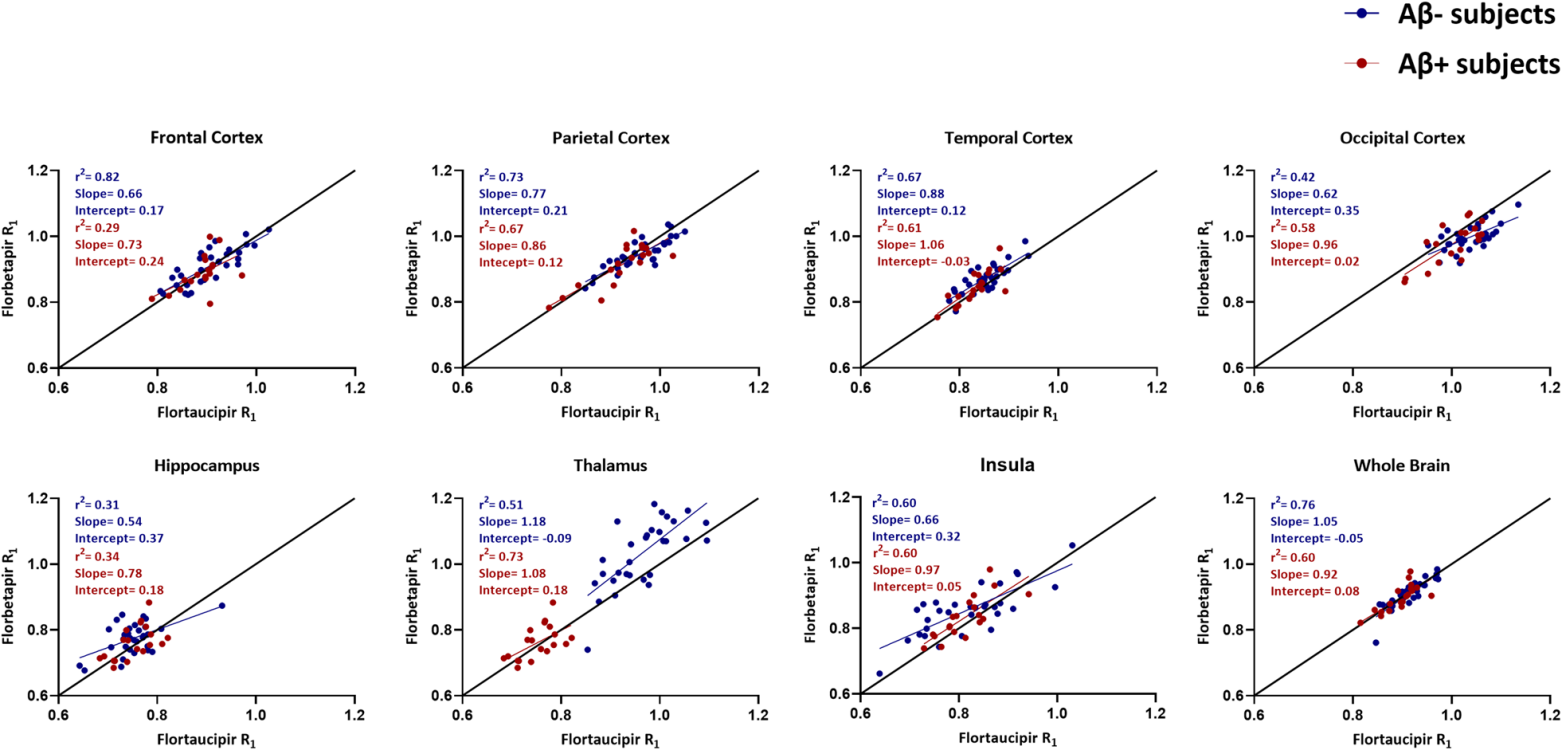
Correlations and slopes obtained from the regional analysis (in native space) between [^18^F]flortaucipir R_1_ and [^18^F]florbetapir R_1_ for Aβ-negative and Aβ-positive subjects

The Aβ-negative group showed significant differences in the parietal cortex, temporal cortex, occipital cortex, hippocampus, thalamus and insula (Fig. 5). Furthermore, significant differences were found in the occipital cortex, thalamus and insula in the Aβ-positive group (Fig. 5). Despite that the R_1_ for some regions was significantly different between the tracers, most of the regions had a good to moderate correlation for R_1_ obtained with [^18^F]florbetapir and [^18^F]flortaucipir (Fig. 6). Also for the Aβ-positive group, most of the regions had a good to moderate correlation for R_1_ obtained with [^18^F]florbetapir and [^18^F]flortaucipir (Fig. 6).

The ICC for each brain region (in native space) is presented in Table 1, separately for the Aβ-negative and Aβ-positive subjects. Most of the regions had an excellent ICC > 0.80; however, the smaller regions had somewhat lower ICC < 0.70.

**Table 1:** ICC between regional [^18^F]flortaucipir R_1_ and [^18^F]florbetapir R_1_ (in native space)_,_ separately for Aβ-negative and Aβ-positive subjects

	ICC	
	Aβ negative	Aβ positive
Frontal cortex	0.90	0.70
Parietal cortex	0.91	0.90
Temporal cortex	0.85	0.83
Occipital cortex	0.62	0.81
Hippocampus	0.67	0.72
Thalamus	0.66	0.81
Insula	0.84	0.84
Putamen	0.65	0.85
Brainstem	0.61	0.32
Whole brain	0.92	0.87

## Discussion

The current study made a head-to-head comparison between R_1_ obtained from two different dynamic PET tracers. In line with previous studies [13, 19], the results of the current study showed that RPM R_1_ derived from dynamic [^18^F]florbetapir and [^18^F]flortaucipir PET scans were well correlated with each other irrespective of the small clusters (range 0.2–2.6% of the whole brain) with significant differences observed during voxel-wise comparisons. In a previous head-to-head comparison study, it has been demonstrated that R_1_ estimates from a [^18^F]florbetapir PET scan strongly correlated with CBF obtained from [^15^O]H_2_O PET [17]. Based on the results of the current study and the study by Ottoy et al. (2019) [17], one could state that R_1_ may serve as a surrogate for rCBF, for both [^18^F]flortaucipir and [^18^F]florbetapir (particularly for cortical regions). The R_1_ obtained with [^18^F]florbetapir and [^18^F]flortaucipir PET are of clinical interest as amyloid and tau tracers benefit from the ability to provide information on both rCBF and Aβ pathology or tau pathology in one imaging session, respectively. Most of the cortical regions had no significant differences in R_1_ between the tracers for both Aβ-negative and Aβ-positive SCD subjects. Despite the significant clusters, the R_1_ obtained from [^18^F]florbetapir and [^18^F]flortaucipir were well correlated (*r*^2^ > 0.5). These findings were supported by the results of the regional analysis in native space.

Significantly higher R_1_ values in the hippocampus for [^18^F]flortaucipir were observed when compared to [^18^F]florbetapir in both Aβ-negative and Aβ-positive SCD subjects. Hippocampus is a region that is crucial in AD with respect to tau studies, since the involvement of the hippocampus is thought to occur at a critical stage of tau pathology progression [29]. [^18^F]flortaucipir has been characterized by off-target binding in the basal ganglia, thalamus and choroid plexus [30–32]. The off-target binding in the choroid plexus is of particular interest since it may cause spill-in to the anatomically near hippocampus. This spill-in effect may lead to an artificially increased [^18^F]flortaucipir activity in the hippocampus which leads to an inaccurate quantification of hippocampal tau load. The choroid plexus consists of a dense collection of capillaries in an ependymal stroma surrounded by a layer of epithelium [33]. The choroid plexus of the lateral ventricles may have several structures that could bind to [^18^F]flortaucipir, including melanin [34], calcification [35], Biondi bodies [36, 37] and iron deposits [38]. Off-target binding is problematic as the hippocampus is among the earliest regions affected by tau pathology and accurate assessment of tau accumulation in this region is important in the understanding of the natural time course of AD [29]. Earlier studies have investigated methods to reduce the spill-in effect of the choroid plexus, such as eroding voxels of the hippocampus [39], various partial volume correction methods [39–41] and linear regression approaches [39, 40]. These techniques led to decreased correlation between hippocampus and choroid plexus tracer binding, presumably due to decreasing the spill-in effects [39–42]. The elevated [^18^F]flortaucipir signal in the hippocampus when compared to [^18^F]florbetapir could be partly explained by spill-in from the off-target binding in the choroid plexus.

Higher R_1_ values were observed for [^18^F]florbetapir in part of the thalamus (bilateral) when compared to [^18^F]flortaucipir in both Aβ-negative and Aβ-positive SCD subjects. The thalamus mainly consists of GM; however, it also contains two thin layers of white matter (WM), including the stratum zonale that covers the dorsal surface, and the external and internal medullary laminae [43]. [^18^F]florbetapir has previously shown non-specific binding in WM [44]. WM mainly consists of myelin, which is highly lipidic. The lipophilic character of [^18^F]florbetapir presumably explains the non-specific binding in this region [45, 46]. In our study, the number of voxels that were significant in the thalamus for WM was ± 863 voxels for Aβ-negative SCD subjects, accounting for 55.4% of total voxels in the WM thalamus and ± 500 voxels (31.5% of total voxels in the WM thalamus) for Aβ-positive SCD subjects, while the significant number of voxels in the GM for this region was much lower (Aβ−: ± 211 voxels: 24.1% of total GM voxels, Aβ+: ± 155 voxels; 17.7% of total GM voxels). This may be a possible explanation for the observed significant differences in R_1_ between [^18^F]florbetapir and [^18^F]flortaucipir in the thalamus in this study.

A decrease in rCBF in the thalamus in case of Aβ-positive SCD subjects (as shown in Fig. 6, subplot associated with Thalamus) could be because of the involvement of the Papez circuit in the memory function. The Papez circuit is an anatomical circuit which starts and ends in the hippocampus [47]. Earlier studies have shown that lesions in any part of this circuit can cause memory dysfunction [48–50]. It is not uncommon that there is decreased cerebral blood flow in the thalamus in Aβ-positive SCD subjects [51]. Kobayashi et al. [51] also observed decreased thalamic blood flow in mild AD patients using quantitative brain perfusion SPECT. The researchers concluded that lower rCBF in the thalamus might be the result of a remote metabolic effect in the Papez circuit.

Almost all other GM cortical regions showed similar R_1_ values for both tracers. Besides that, the R_1_ values obtained from [^18^F]flortaucipir and [^18^F]florbetapir showed good correspondence in the voxel-wise analysis and in the regional analysis (Figs. 4, 6), indicating that R_1_ may be considered as a reliable measure of cortical rCBF irrespective of tracer choice. The smaller brain regions, such as the insula and hippocampus, had weaker correlations in R_1_ between the two tracers, and this could be explained by the fact that smaller regions are more prone to noise. Furthermore, some ROIs showed significant differences in the Aβ-negative subjects that were not present in the Aβ-positive group. No clarification could be found for these results. Therefore, further research, aimed to address this issue, is necessary to determine the clinical implications. Despite different kinetics, the present study showed that R_1_ obtained from [^18^F]florbetapir was comparable with [^18^F]flortaucipir R_1_ in the cortical regions. Furthermore, the TRT of R_1_ is better for both tracers, 2.1 ± 1.1% and 1.8 ± 1.3% [8, 18], respectively, when compared to the TRT of perfusion as measured with [^15^O]H_2_O PET (9.7 ± 10.4%) [8]. K_1_ estimates obtained from [^15^O]H_2_O PET presented a lower test–retest repeatability, possibly due to day to day intrasubject variabilities (that are not related to any underlying pathology). Since the intrasubject differences (non-pathological) remain more or less constant throughout the brain, normalizing the K_1_ to a reference region K_1_’ (i.e. R_1_) could correct for these differences and improve the TRT.

One of the limitations of the current study is that it was performed only in SCD subjects. It should preferably be reevaluated in patient groups, especially if pathological alterations in rCBF are expected such as in neurodegenerative disorders. For this purpose, the gold standard, [^15^O]H_2_O PET can be considered to evaluate direct CBF in first place. Furthermore, no partial volume correction method was applied on the data, which can eliminate the spill-in effects from the choroid plexus and the WM. Yet, as both tracer studies were collected on the same PET system and used the same reconstruction methods and settings, both datasets have a matched spatial resolution. It is important to acknowledge that partial volume effects (PVE) depend on the contrast between regions, influenced by both tracer and uptake time. Consequently, PVE may not be the same across the tracers under examination. However, R_1_ is primarily determined by the early uptake phase of the tracer (< 100 s). Therefore, we anticipate that the influence of PVE on R_1_ may be similar for both tracers. Nonetheless, certain distinctions, such as possible spill-in from the choroid plexus and white matter, cannot be definitively dismissed. Additionally, validation of the use of R_1_ as cortical rCBF surrogate was evaluated for [^18^F]florbetapir and [^18^F]flortaucipir and it is essential to note that the extension of these results to other tracers requires further assessments.

## Conclusion

The present study showed that [^18^F]flortaucipir and [^18^F]florbetapir R_1_ values were well correlated in GM cortical regions. Put together with previous findings by Ottoy et al. (2019) [17], a cautious claim can be made that R_1_ values of both tracers are a valid marker for cortical rCBF. However, the use of R_1_ as a tracer-independent rCBF surrogate should be performed with caution in case of some non-cortical areas, as shown in this study. Furthermore, generalization to other tracers is not directly implied by this study, since each new PET tracer needs thorough validation.

## Data Availability

The datasets used and/or analysed during the current study are available from the corresponding author on reasonable request.

## Abbreviations

Aβ: Amyloid beta
AD: Azheimer’s disease
CBF: Cerebral blood flow
FDG: [^18^F]-fluoro-2-deoxy-d-glucose
FWE: Family-wise error
ICC: Intraclass correlation coefficient
MNI: Montreal Neurological Institute
MRI: Magnetic resonance imaging
PET: Positron emission tomography
*r*^2^: Correlation coefficient
rCBF: Relative cerebral blood flow
ROIs: Regions of interest
RPM: Receptor parametric mapping
SCD: Subjective cognitive decline
SPM12: Statistical Parametric Mapping 12
SRTM: Simplified reference tissue model
SUVr: Standardized uptake value ratio
TAC: Time activity curve
TRT: Test–retest repeatability
